# Frequency Modulation Control of an FMCW LiDAR Using a Frequency-to-Voltage Converter

**DOI:** 10.3390/s23104981

**Published:** 2023-05-22

**Authors:** Jubong Lee, Jinseo Hong, Kyihwan Park

**Affiliations:** Department of Mechanical Engineering, Gwangju Institute of Science and Technology (GIST), 123, Cheomdangwagi-ro, Buk-gu, Gwangju 61005, Republic of Korea

**Keywords:** frequency-modulated continuous-wave (FMCW) LiDAR, frequency control, range finding

## Abstract

An FMCW LiDAR (frequency-modulated continuous-wave light detection and ranging) is a sensor that can measure distance using optical interference frequency ($f_{b}$). This sensor has recently attracted interest because it is robust to harsh environmental conditions and sunlight due to the wave properties of the laser. Theoretically, when the frequency of the reference beam is linearly modulated, a constant $f_{b}$ is obtained with respect to the distance. However, when the frequency of the reference beam fails to be linearly modulated, the distance measurement is not accurate. In this work, linear frequency modulation control using frequency detection is proposed to improve the distance accuracy. The FVC (frequency to voltage converting) method is used to measure $f_{b}$ for high-speed frequency modulation control. The experimental results show that linear frequency modulation control using an FVC improves FMCW LiDAR performance in terms of control speed and frequency accuracy.

## 1. Introduction

A pulsed TOF LiDAR (time-of-flight light detection and ranging) obtains the distance of an object by calculating the timing difference of an emitted pulse signal and the received signal [1]. It is sensitive to environmental factors such as rain, snow, and fog because the emitted pulse laser can be diffracted by the particles of snow and rain, which causes severe measurement errors. In order to overcome these problems, several approaches were applied, such as multi-echo and radius outlier removal methods [2]. The FMCW (frequency-modulated continuous wave) LiDAR has recently been of interest in the industrial fields of mixed reality(MR)/augmented reality(AR) autonomous ground vehicles, unmanned aerial vehicles, and defense armour because it is robust to the harsh environmental conditions owing to the wave properties of the laser [3,4]. Hence, the laser beam can propagate well through the rain, snow, and fog [5]. Additionally, it can measure the speed of an object by the Doppler effect.

An FMCW LIDAR obtains distance by calculating the optical interference frequency ($f_{b}$) generated by interfering a reference beam whose frequency is modulated with a received beam (object beam) that is reflected from an object [6,7,8]. A tunable laser can be used for modulating the frequency of the laser beam. When a current is applied to a tunable laser, the wavelength of the laser beam is proportionally varied. However, because of the nonlinear relationship between the wavelength and frequency, a linear frequency modulation control is not easy to directly implement [9,10]. Theoretically, when the frequency of the reference beam is linearly modulated, a constant $f_{b}$ is obtained with respect to the distance. However, when the frequency of the reference beam fails to be linearly modulated, the distance measurement is not accurate and differs with respect to time [11].

The linear frequency modulation control of the FMCW LiDAR has been studied using a phase-locked loop (PLL) technique with the pre-distortion method [12,13]. If phase variation is continuous, phase detection can be used instead of frequency detection because the phase obtained by integrating the frequency has the advantage of low jitter and high sensitivity [14,15,16,17,18,19]. However, it has a 2π ambiguity problem which complicates the feedback control configuration. To solve this problem, a pre-distortion method is often used together to avoid the 2π ambiguity problem under the assumption that the pre-distortion method is robust to environmental variation so that the phase change is less than 2π.

Linear frequency modulation control using frequency detection is proposed to improve the distance accuracy. Since the proposed method uses a frequency detector, it can be used for a large variation of $f_{b}$. In addition, it has the advantage of being robust to the external environmental change because $f_{b}$ is controlled using a feedback method. Conventionally, FFT (fast Fourier transform) is the most typical method to measure frequency. It has the strong advantage of high accuracy [20]. However, the FFT needs digital signal processing, which takes time to measure frequency, so it is not suitable for feedback control. In this work, the FVC (frequency-to-voltage converting) method is used instead of the FFT to measure $f_{b}$ for high speed frequency modulation control [21,22]. The FVC method causes a ripple noise due to the one-shot converter, which requires the signal processing of a root-mean-square to reduce it [23]. Hence, it is necessary to perform a mathematical analysis of the FVC to trade between the ripple noise and control stability. The experimental results show that linear frequency modulation control using an FVC improves an FMCW LiDAR’s performance in terms of control speed and frequency accuracy.

## 2. FMCW LiDAR System

### 2.1. Principle of Distance Measurement in FMCW LiDAR

**Figure 1:**
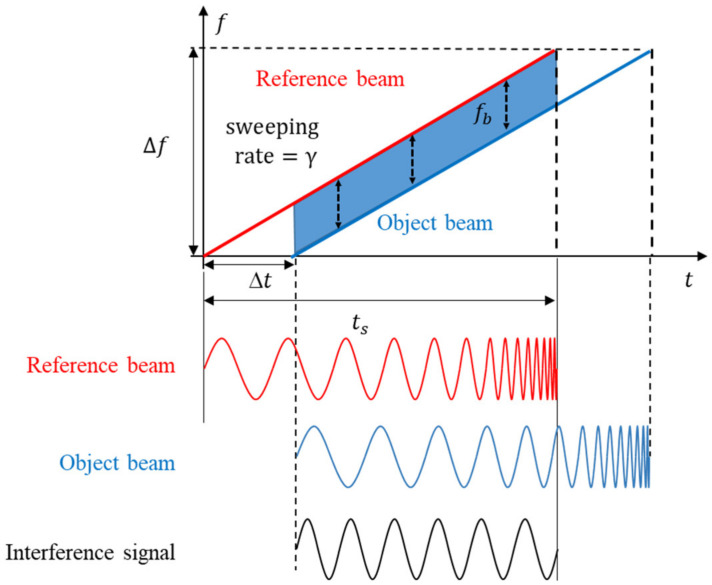
Principle of distance measurement in FMCW LiDAR.

Figure 1 shows the principle of distance measurement in a frequency-modulated continuous-wave (FMCW) LiDAR. When the frequency of the reference beam is linearly modulated up to the frequency, Δf, during the sweeping time, $t_{s}$, the reflected beam is delayed by the time difference, Δt. Here, Δt is proportional to the distance to the object. When those beams are interfered, the interference frequency of the interference signal, $f_{b}$, is obtained. Then, Δt is indirectly obtained by $f_{b}$ using the triangle similarity condition.

The sweeping rate of the linear frequency modulation, γ, defined as $\frac{\Delta f}{t_{s}}$ can also be represented by $f_{b}$ and Δt as:

(1) $$\gamma =\frac{\Delta f}{t_{s}}=\frac{f_{b}}{\Delta t}$$

Hence, the distance, d, between LiDAR and the object, *d*, is written by using Equation (1) as follows:

(2) $$d=\frac{c\cdot \Delta t}{2}=\frac{c\cdot f_{b}}{2\gamma }$$

Therefore, d can be measured using $f_{b}$ instead of Δt, differing from the conventional pulsed TOF LiDAR. The distance resolution, Δd, can be obtained using Equations (1) and (2) by recognizing that the minimum $\Delta f_{b}$ to be measured within the sweeping time is $\frac{1}{t_{s}}$ as follows:

(3) $$\begin{array}{l} \Delta d=\frac{c\cdot \Delta f_{b}}{2\gamma }=\frac{c\cdot \frac{1}{t_{s}}}{2\frac{\Delta f}{t_{s}}}\\ =\frac{c}{2\Delta f} \end{array}$$

The reference beam, $\vec{E_{1}}$, is theoretically represented as follows:

(4) $$\vec{E_{1}}=E_{1}\cos\left(f_{1}t+\phi_{1}\right)$$

where $E_{1}$, $f_{1}$, and $\phi_{1}$ are the amplitude, frequency, and phase of $\vec{E_{1}}$, respectively. The received beam delayed by Δt, $\vec{E_{2}}$ is:

(5) $$\vec{E_{2}}=E_{2}\cos\left(\left(f_{1}-\gamma \Delta t\right)t+\phi_{2}\right)$$

where $E_{2}$, $f_{2}$, and $\phi_{2}$ are the amplitude, frequency, and phase of $\vec{E_{2}}$, respectively. The intensity of the interfered beam I of $\vec{E_{1}}$ and $\vec{E_{2}}$ is:

(6) $$\begin{array}{l} I=E_{1}^{2}+E_{2}^{2}\\ +E_{1}E_{2}\left(\cos\left(2f_{1}-\gamma \Delta t\right)t+\phi_{1}+\phi_{2}\right)\\ +cos\left(\left(\gamma \Delta t\right)t+\phi_{1}-\phi_{2}\right) \end{array}$$

Equation (7) can be rearranged using the trigonometric relation as:

(7) $$\begin{array}{l} I=E_{1}^{2}+E_{2}^{2}\\ +2E_{1}E_{2}\left(\left(\cos\left(f_{1}-\gamma \Delta t\right)t+\phi_{1}\right)\cdot \cos\left(f_{1}t+\phi_{2}\right)\right) \end{array}$$

The DC offset in Equation (7) can be easily eliminated by using a high pass filter. The first cosine term is terahertz frequency; thus, it cannot be measured by a photo detector. Therefore, only the second cosine term is measured and a final interfered beam signal $I_{s}$ can be obtained under the assumption of $\phi_{1}-\phi_{2}=0$ and Equation (1) as:

(8) $$I_{s}=cos\left(f_{b}t\right)$$

A frequency detection sensor can be used to measure $f_{b}$ from $I_{s}$

### 2.2. Composition of FMCW LiDAR

**Figure 2:**
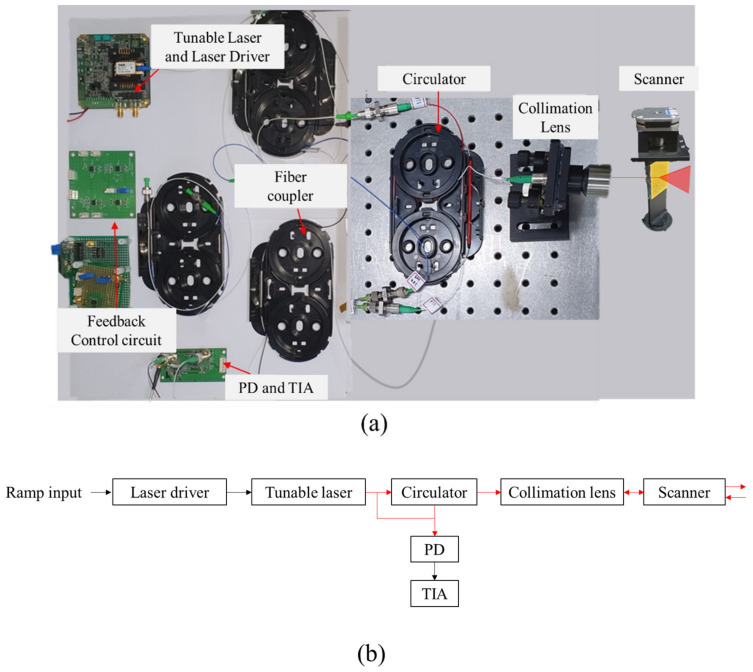
(**a**) Composition of an FMCW LiDAR, (**b**) block diagram of an FMCW LiDAR.

Figure 2 shows the overall composition of the FMCW LiDAR. The overall configuration is largely divided into a laser source unit, an interferometer, a scanner, and a signal processing part. Firstly, the laser source unit includes a tunable laser (SFL1550, Thorlabs, Newton, NJ, USA) and a laser driver (CTL101, Koheron, Princeton Junction, NJ, USA) for generating a frequency-modulated laser beam. A tunable laser can take advantage of the easy modulation of the laser frequency using the current, a long coherence length, and a wide dynamic range of frequencies. These kinds of advantages are suitable for an FMCW LiDAR, which needs a wide frequency bandwidth and long coherence length [24]. Secondly, the interferometer comprises a fiber coupler and circulator (CIR1550, Thorlabs), where reference and object beams are interfered. A reference beam is transmitted and an object beam is received through the collimation lens (F810APC-1550, Thorlabs) and the scanner, respectively. A photo detector (PD) (FGA01FC, Thorlabs) converts the optically interfered beam into current. The voltage is obtained using a trans-impedance amplifier (TIA). Finally, the interference frequency is measured by a frequency detector in the electronic circuits. Figure 2b shows the block diagram of an FMCW LiDAR.

## 3. Frequency Modulation of a Tunable Laser

**Figure 3:**
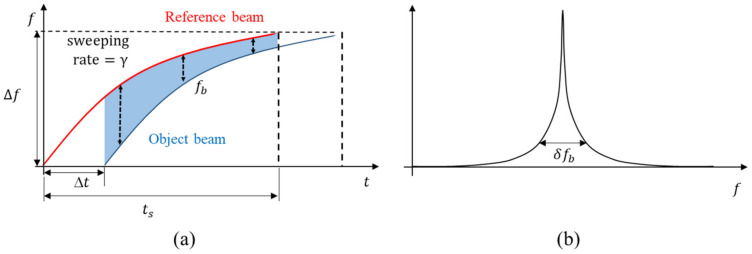
(**a**) Nonlinear frequency sweeping and its effect on interference signal, (**b**) frequency spectrum of $\delta f_{b}$.

A tunable laser can be used for modulating the laser beam frequency because the laser wavelength proportionally increases with the current. Thus, the frequency of the laser is inversely proportional to the applied current. Hence, realizing a linear frequency modulation by only applying the current to the tunable laser is almost impossible. Therefore, when the current is increased proportionally, the interference frequency, $f_{b}$, varies even at the same distance, considering time as shown in Figure 3a, which causes inaccuracy in the distance measurement. The large variance of $f_{b}$ is well observed with a wide frequency spectrum, $\delta f_{b}$, as shown in Figure 3b. Hence, a linear frequency modulating control of the tunable laser is required.

## 4. Proposed Control Method of Linear Frequency Modulation

**Figure 4:**
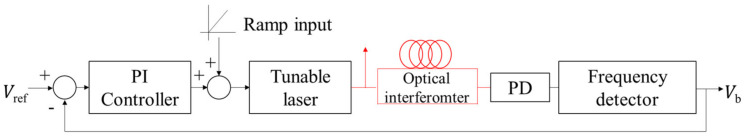
Configuration block diagram of frequency modulation control.

The frequency of the interference signal at a known distance is controlled using an additional optical interferometer installed internally, as shown in Figure 4, to solve the problem. Herein, the additional fiber optical interferometer is constructed with an optical path difference between the reference and object beams. The corresponding interference frequency of the two beams is then detected at PD and is converted into voltage, $V_{b}$, proportional to it at the frequency detector. This frequency is used for a desired frequency, $V_{ref}$, of feedback control, as shown in Figure 4.

The tunable laser is a plant to be controlled. A ramp input is added to the PI controller output to provide the linear frequency sweeping signal. The beam frequency, which is emitted from tunable laser, can be linearly modulated when the frequency of the tunable laser is successively controlled.

A frequency detector is used for obtaining $f_{b}$ as a sensor. Conventionally, the FFT is the most typical method to measure frequency due to its strong advantage of high accuracy. However, the FFT needs digital signal processing, which takes time to measure a frequency. A high-speed frequency detector is required for real-time feedback control because the FFT method is not suitable for real-time feedback control. Instead of the FFT, the FVC is used in the current study to measure the interference frequency for feedback control. However, the FFT method is used to measure 3D images of an object because the real-time calculation is unnecessary for calculating the interference frequency, which results in measurement accuracy.

## 5. Design of an FVC for Feedback Control

**Figure 5:**
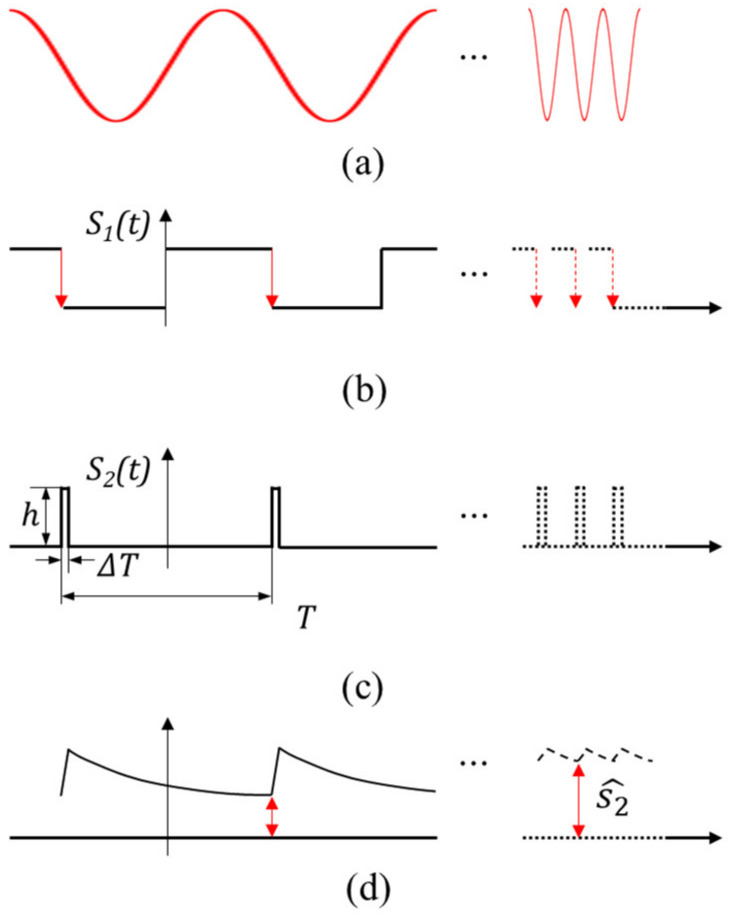
(**a**) Interference signal, (**b**) square wave signal $s_{1}\left(t\right)$ converted from interference signal, (**c**) pulse train signal $s_{2}\left(t\right)$, (**d**) output of FVC includes ripple noise.

The FVC is largely divided into three steps. Firstly, the interference signal in Figure 5a is supposed to be converted to the square wave, $s_{1}\left(t\right)$, using a Schmitt trigger, as shown in Figure 5b. Secondly, at the falling edges of $s_{1}\left(t\right)$, the signal is converted to the very short pulse train, $s_{2}\left(t\right)$, using a one-shot converter, as shown in Figure 5c. Herein, $s_{2}\left(t\right)$ has the constant pulse width regardless of the input frequency.

The principle of the FVC is based on the proportional average value of $s_{2}\left(t\right),$ $\hat{s_{2}}$ to the frequency of $s_{2}\left(t\right)$, f, which is mathematically proven as follows:

(9) $$\hat{s_{2}}=\frac{1}{T}\int_{-\frac{T}{2}}^{\frac{T}{2}}s_{2}\left(t\right)dt=fh\Delta T$$

where T is the period of $s_{2}\left(t\right)$, f is the frequency of $s_{2}\left(t\right)$, h is the amplitude of $s_{2}\left(t\right)$, and ΔT is the pulse width of $s_{2}\left(t\right)$.

Notably, $s_{2}\left(t\right)$ can be expressed by using the Fourier series as

(10) $$s_{2}\left(t\right)=a_{0}+\overset{\infty }{\underset{k=1}{\sum }}(a_{k}cos\frac{2\pi kt}{T}+b_{k}sin\frac{2\pi kt}{T})$$

where $a_{0}=\frac{1}{T}\int_{-\frac{T}{2}}^{\frac{T}{2}}s_{2}\left(t\right)dt$, $a_{k}=\frac{2}{T}\int_{-\frac{T}{2}}^{\frac{T}{2}}s_{2}\left(t\right)cos\frac{2\pi kt}{T}dt$, and $b_{k}=\frac{2}{T}\int_{-\frac{T}{2}}^{\frac{T}{2}}s_{2}\left(t\right)sin\frac{2\pi kt}{T}dt$.

Equation (10) shows that $a_{0}$ is expressed as the same as $\hat{s_{2}}$. Hence, the average of the function, $s_{2}\left(t\right)$, can be obtained by applying the low pass filter (LPF) of $s_{2}\left(t\right)$ because the high orders of sinusoidal functions are substantially reduced when the cutoff frequency of an LPF is low. However, ripple noise must exist due to the non-eliminated high orders of sinusoidal functions. The cutoff frequency of LPF should be lowered to reduce the magnitude of the ripple noise. However, when the LPF with a low cutoff frequency is used for frequency feedback control, a large phase delay can cause instability in a feedback system. Therefore, a tradeoff must exist between the magnitude of the ripple noise of the FVC and the control stability.

**Figure 6:**
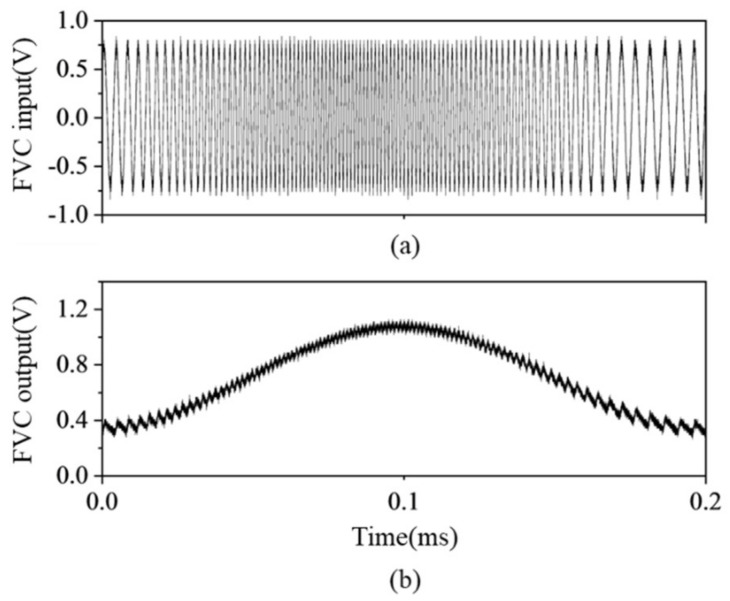
(**a**) Input frequency modulation signal of FVC, (**b**) response of FVC frequency detection.

For example, when the interference frequency is varied from 250 kHz to 550 kHz, as shown in Figure 6a, the FVC output varied proportionally to the frequency, as shown in Figure 6b. In the output of the FVC, ripple noise is observed with different amplitudes depending on the frequency. A large interference frequency indicates a ripple noise with a small amplitude. The ripple noise has magnitudes of 150 and 100 mV when the input frequencies are 250 and 550 kHz, which are measured at short and long distances, respectively. The experiment result indicates that the ripple noise can be reduced by increasing the interference frequency, which is obtained using a long optical path difference of the additional fiber interferometer shown in Figure 2.

## 6. Experimental Results of Frequency Control

**Figure 7:**
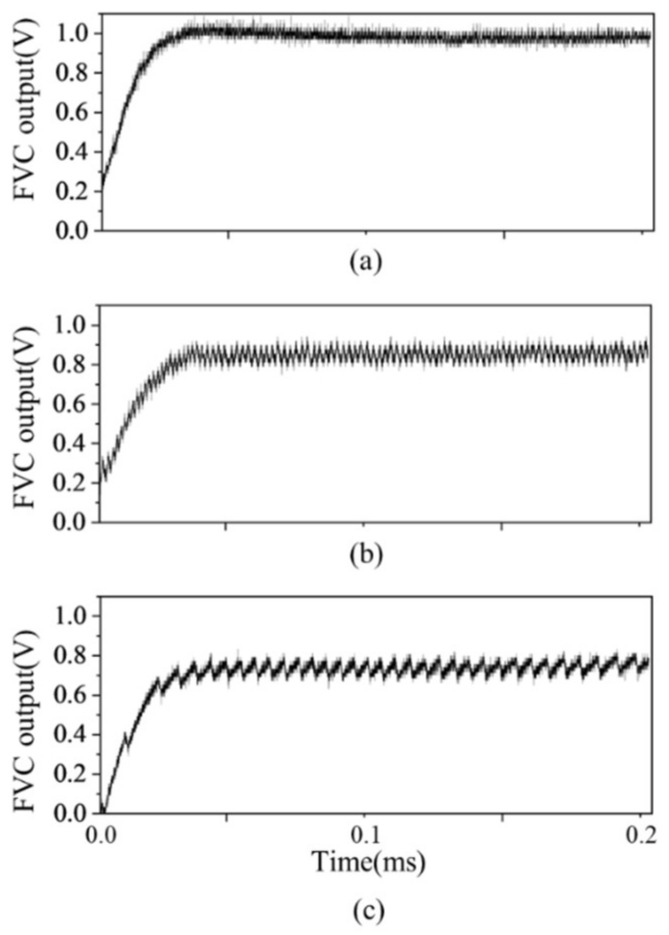
Output signal at FVC when $f_{b}$ is (**a**) 900 kHz, (**b**) 550 kHz, and (**c**) 250 kHz.

The control experiment is performed under the following conditions: sweeping frequency Δf=4.2 GHz, sweeping rate γ=21 GHz/ms, and sweeping time $t_{s}=200\;\mu s$. Figure 7 shows that the frequency control can be used with large $f_{b}$ changes. To show the frequency control performance in the change in $f_{b}$, the experiment was conducted by changing $f_{ref}$ from 250 to 900 kHz. The change in $f_{ref}$ was simply made by changing $V_{ref}$, which is the input signal of the control. Figure 7a shows that the FVC output converges to 1 V when the $f_{ref}$ is set to 900 kHz. The ripple noise is smaller than the electronic noise and cannot be measured. Figure 7b shows that the FVC output converges to 830 mV when $f_{ref}$ is 550 kHz, the same as $f_{b}$. The FVC output includes a ripple noise with a magnitude of 100 mV and frequency of 550 kHz. Figure 7c shows that the FVC output converges to 720 mV when $f_{ref}$ is 250 kHz. The FVC output includes a ripple noise with a magnitude of 150 mV and frequency of 250 kHz. Consequently, as $f_{ref}$ changes from 250 to 900 kHz, the FVC output increases from 720 mV to 1 V when frequency control is performed. The ripple noise decreases as $f_{b}$ increases.

**Figure 8:**
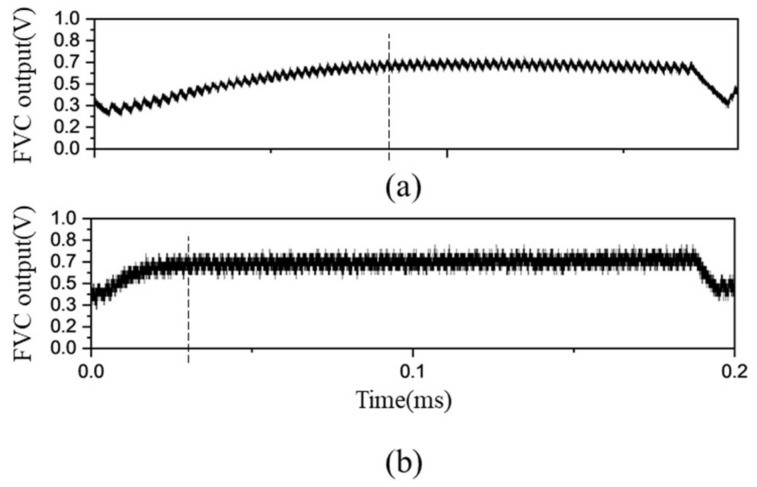
(**a**) Output signal at FVC with low cutoff frequency, (**b**) output signal at FVC with high cutoff frequency.

Figure 8a shows the FVC output signal when an LPF with a low cutoff frequency $\left(10\;\mathrm{kHz}\right)$ is used. The signal includes approximately 50 mV of ripple noise. The FVC output reaches a steady state at 80 μs. When an LPF with a high cutoff frequency (40 kHz) is used, the ripple noise increased to 100 mV, as shown Figure 8b. However, the FVC output reaches the steady state at a fast time in 30 μs. This result is attributed to decreased phase delay of the LPF. Therefore, a tradeoff must exist between the ripple noise magnitude of the FVC and the control bandwidth.

**Figure 9:**
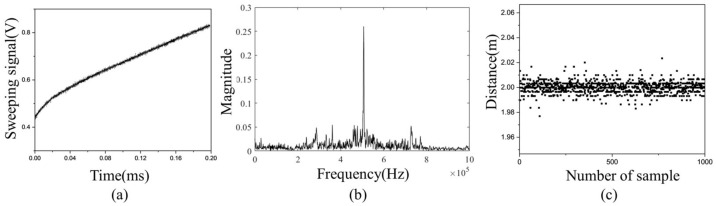
(**a**) Frequency modulation signal, (**b**) FFT, and (**c**) measured distance when frequency control is appled.

**Figure 10:**
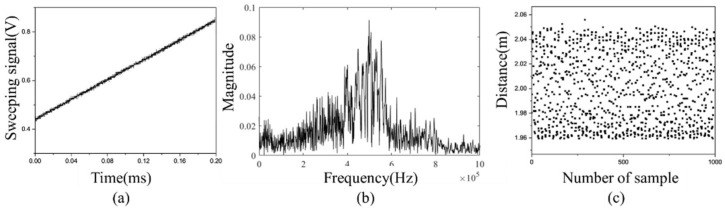
(**a**) Frequency modulation signal, (**b**) FFT, and (**c**) measured distance when frequency control is not appled.

The distance and frequency accuracy improvement with and without control was confirmed through experiments. Figure 9a is a frequency modulation signal which is distorted through frequency modulation control. The signal increases nonlinearly from 420 to 810 mV over a period of 0.2 ms. Figure 9b shows the result of an FFT analysis of the internal interference frequency with the control. The interference frequency with the control has a peak frequency of 550 kHz with a FWHM (full width at half maximum) of 30 kHz. Figure 9c shows that the measured distance has an average of 2 m and an error of 3 cm when an object at a distance of 2 m is measured 1000 times by transmitting a controlled beam. On the other hand, Figure 10a shows that the frequency modulation signal is a ramp input that increases from 420 to 820 mV when the control is not performed. The uncontrolled internal interference frequency shown in Figure 10b has a peak frequency of 550 kHz and a FWHM of 150 kHz. Figure 10c shows that when 2 m distance is measured by transmitting an uncontrolled beam, the measured distance has an average of 2.01 m and an error of 10 cm. Consequently, the frequency accuracy and distance accuracy increases by a factor of 5 when frequency control is performed.

**Figure 11:**
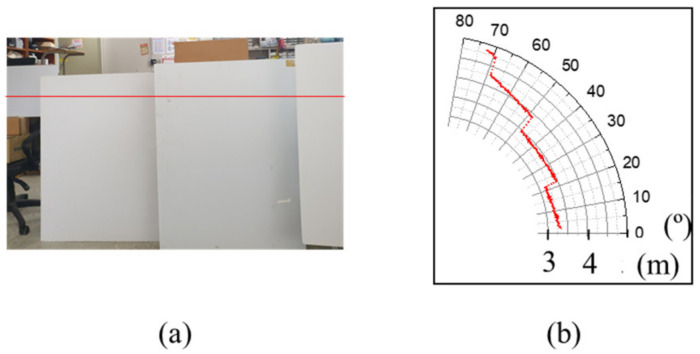
(**a**) Setup of FMCW LiDAR scanning situation, (**b**) polar coordinate graph of scanning image with control.

A 2D-scan experiment is conducted with the applied controller. The experiment was conducted under the conditions of a measurement angle of 70°, a scan speed of 1 Hz, and 1000 measurement points. The interference frequency was analyzed through an FFT and calculated for distance measurement. Figure 11a shows the distance of objects located between 3 and 5 m. Figure 11b shows the 2D scan image using a polar coordinate.

## 7. Conclusions

In the field of FMCW LiDARs, the inaccurate interference frequency can be detected because of the specification of the laser source or environmental disturbance, such as temperature change. Linear frequency modulation control is applied by feeding back the internal interference frequency to solve the problem. The interference frequency is detected using an FVC method, which enables fast frequency detection while performing linear frequency sweeping control. The experimental results show that frequency control using an FVC can be applied to an FMCW LiDAR to improve distance accuracy, which is validated by comparing the FWHM of frequency based on the FFT method.

## Data Availability

The data presented in this study are available on request from the corresponding author. The data are not publicly available due to institutional confidentiality provisions.
